# Inflammatory cytokine and microRNA responses of primary human dendritic cells cultured with *Helicobacter pylori* strains

**DOI:** 10.3389/fmicb.2013.00236

**Published:** 2013-08-20

**Authors:** Anaïs Hocès de la Guardia, Cathy Staedel, Itidal Kaafarany, Aurélien Clément, Claire Roubaud Baudron, Francis Mégraud, Philippe Lehours

**Affiliations:** ^1^Bacteriology Laboratory, Université BordeauxBordeaux, France; ^2^Institut National de la Santé et de la Recherche Médicale, U853Bordeaux, France; ^3^ARNA Laboratory, Université BordeauxBordeaux, France; ^4^Institut National de la Santé et de la Recherche Médicale, U869Bordeaux, France; ^5^Pôle de Gérontologie Clinique, CHU de BordeauxBordeaux, France

**Keywords:** *Helicobacter pylori*, dendritic cell, inflammation, TNFα, microRNA

## Abstract

Primary human dendritic cells (DC) were used to explore the inflammatory effectors, including cytokines and microRNAs, regulated by *Helicobacter pylori*. In a 48 h *ex-vivo* co-culture system, both *H. pylori* B38 and B45 strains activated human DCs and promoted a strong inflammatory response characterized by the early production of pro-inflammatory TNFα and IL-6 cytokines, followed by IL-10, IL-1β, and IL-23 secretion. IL-23 was the only cytokine dependent on the *cag* pathogenicity island status of the bacterial strains. DC activation and cytokine production were accompanied by an early miR-146a upregulation followed by a strong miR-155 induction, which mainly controlled TNFα production. These results pave the way for further investigations into the nature of *H. pylori* antigens and the subsequently activated signaling pathways involved in the inflammatory response to *H. pylori* infection, the deregulation of which may likely contribute to gastric lymphomagenesis.

## Introduction

*Helicobacter pylori* infection causes a chronic gastric mucosal inflammation, leading to peptic ulcer disease in 5–10% of the infected people, and to gastric adenocarcinoma and low-grade mucosa-associated lymphoid tissue (MALT) lymphoma in 1% of the cases. The pathogenesis of *H. pylori* infection has been linked to the strength of inflammation it promotes, which is correlated to the functionality of the *cag* pathogenicity island (PAI) in the bacteria (Censini et al., 1996). Gastric epithelial cells constitute the first line of defense against *H. pylori*; they produce interleukin (IL)-8, which promotes the recruitment of polynuclear cells. Immune cells including macrophages, dendritic cells (DC) and mucosa infiltrating lymphocytes take part in the innate and adaptative immune responses to the bacteria.

The pro-inflammatory properties of *H. pylori* strains have been mainly evaluated by measuring IL-8 production of the AGS gastric epithelial cell line, but they deserve to be investigated on immune cells due to their orchestrated pro- and anti-inflammatory cytokine production in response to pathogens. *In vivo* the professional antigen-presenting DC were found to be recruited into the gastric mucosa and, due to the emission of dendrites inserting themselves between epithelial cell tight junctions, they may be able to interact with *H. pylori* (Rescigno et al., 2008; Shiu and Blanchard, 2013). Their role in *H. pylori*-induced inflammatory response needs to be clarified.

Host defense against pathogens requires the induction of appropriate innate immune responses, as excessive or inappropriate activation of the immune system can be deleterious to the organism. Therefore, various immune regulators, including microRNAs (miRNA), also take part in the immune responses (Baltimore et al., 2008). MiRNA received considerable attention because of their implication in maintaining homeostasis in fundamental biological processes in non-pathological states, and their deregulation in pathological states (Sonkoly and Pivarcsi, 2009). Changes in miRNA expression in response to bacterial infection have been reported, including *H. pylori* infection in the gastric mucosa, in gastric epithelial cells and in immune cells (Zhang et al., 2008; Fassi Fehri et al., 2010; Belair et al., 2011; Matsushima et al., 2011).

In the present study, primary human DCs were used to explore the inflammatory effectors induced by *H. pylori* strains. Two fully sequenced *H. pylori* strains were included for this purpose, i.e., strains B38 (Thiberge et al., 2010) and B45 (Lehours et al., 2011) which are *cag*PAI negative and *cag*PAI positive, respectively. In parallel to cytokine production, miRNA changes induced by *H. pylori* were analyzed; they included measuring miR-146 and miR-155, to which specific immunomodulatory functions in *H. pylori* infection were assigned after performing loss of function experiments.

## Materials and methods

### Ethics statement

Written consent of hemochromatosis patients was obtained and approved by the French Ministry of Research and the French Aquitaine Limousin Blood Bank's (Bordeaux, France) ethics committee (approval number DC-2012-1648).

### *In vitro* generation of DCs

All tissue culture reagents were purchased at Invitrogen (Marly Le Roi, France). Peripheral blood mononuclear cells (PBMC) from hemochromatosis patients were isolated by centrifugation on a Ficoll gradient and captured using magnetic CD14 microbeads (Miltenyi Biotec, Paris, France), according to the manufacturer's protocol. To generate immature DCs, monocytes were grown for 7 days in the presence of GM-CSF (50 ng/ml) and IL-4 (25 ng/ml) in RPMI-1640 medium supplemented with 10% heat-decomplemented fetal calf serum, 2 mM of L-glutamine, and 50 μg/ml of vancomycin (Sandoz, Levallois Perret, France) at 37°C in a 5% CO_2_ atmosphere.

### Culture of *H. pylori* strains

B38 is a *cag*PAI negative strain, harboring the *vac*A s2(i2)m2 allele. B45 is a *cag*PAI positive strain, harboring the *vac*A s1(i1)m1 allele (Lehours et al., 2004b; Thiberge et al., 2010; Lehours et al., 2011). The P12 *H. pylori* strain or its Δ*cag*PAI isogenic deletion mutant (kindly provided by R. Peek, Vanderbilt University, Nashville, TN, USA) as well as 6 *cag*PAI positive and 6 *cag*PAI negative ulcer-associated strains (Lehours et al., 2004a) were also included. All strains were cultivated for 48 h at 37°C under microaerobic conditions (5% O_2_) on selective agar consisting of 21.5 g of Wilkins Chalgren agar, 50 ml of human blood, 10 μg/ml of vancomycin, 10 μg/ml of cefsulodin, 5 μg/ml of trimethoprim, and 10 μg/ml of amphotericin B.

### Co-cultures of immature DCs and *H. pylori* strains

Immature DCs were washed once in PBS and plated onto 24-well plastic plates at a density of 5.10^5^ cells per well in 1 ml of RPMI-1640 growth medium. Bacteria were recovered from the agar plates using a swab and resuspended in RPMI-1640 growth medium at an optical density of 0.6 at 600 nm, which corresponds to 3.10^7^ CFU/ml. The bacteria were added to the DCs at the indicated multiplicity of infection (MOI) 1 and the co-cultures were further incubated at 37°C in a 5% CO2 atmosphere for 48 h.

### Dendritic cell activation and cytokine analysis

The DC surface activation markers CD40, CD80, CD83, CD86, and HLA-DR, as well as the following secreted cytokines, IL-2, IL-4, IL-6, IL-10, and TNFα, were evaluated by flow cytometry using the Human Cytokine Bead Array Th1/Th2/Th17 Kit (Becton Dickinson, Le Pont de Claix, France). IL-8, IL-12, IL-1β, and IL-23 production was assessed by ELISA (Ready–SET–Go, eBioscience, San Diego, CA, USA). DCs alone or stimulated with *Escherichia coli* lipopolysaccharide (LPS) (100 ng/ml) (Sigma Aldrich, St Quentin Fallavier, France) were used as negative or positive controls for DC activation, respectively.

### Dendritic cell apoptosis

The occurrence of DC apoptosis was assessed using annexin V staining and propidium iodure incorporation. DCs were centrifuged at 2000 rpm for 10 min at 4°C, washed once in cold PBS and resuspended in 25 μL of 0.01 M HEPES (pH 7.4), 0.14 M NaCl, and 0.25 mM CaCl_2_. The cells were labeled with 2.5 μL of anti-annexin V antibody (eBioscience) and 1 μL of propidium iodure (1 mg/mL) (Sigma Aldrich) for 15 min at 4°C and analyzed by flow cytometry.

### Oligonucleotide transfection

Transfections were performed in 24-well plates using lipofectamin 2000 (Invitrogen) according to manufacturer's instructions, except that 5 μ l/well were used. DCs were transfected twice (at day −2 and then 2 h before infection) with 100 nM of antisense miRNA (as155 or as146) or scrambled locked-nucleic-acid (LNA)-modified oligonucleotides (Table A1).

### RNA extraction and miRNA quantitative RT-PCR

DCs were collected in Eppendorf tubes and pelleted by centrifugation at 2000 rpm for 5 min at room temperature. Total RNA was extracted from the DC pellet using a Trizol reagent (Invitrogen), according to manufacturer's instructions. RNA concentrations were determined by spectrophotometry (NanoDrop Technologies, Wilmington, DE, USA). RNA quality was determined on an Agilent 2100 Bioanalyzer (Agilent Technologies, Santa Clara, CA, USA). For miRNA quantitations, 250 ng of total RNA were retrotranscribed using the miScript Reverse Transcription kit (Qiagen, Courtaboeuf, France) and qPCR was performed using a SYBr Green PCR kit and hsa-miR-155, hsa-miR-146a and SNOR25 specific primers (Qiagen), according to the manufacturer's instructions. The amplification profiles were measured on a Stratagene Mx3005P instrument (Life Technologies, Saint Aubin, France).

### Statistical analysis

Data were expressed as a ratio of fold induction compared to non-activated DCs. For each experiment, all experimental conditions were tested three times and each experiment was conducted at least three times for each strain. Statistical analyses were done using the non-parametric Mann-Whitney test. A *p*-value of less than 0.05 was considered significant. All statistics were performed using SPSS 16.0F for Windows software (SPSS Inc., Chicago, IL, USA).

## Results

### Dendritic cell activation by *H. pylori*

**Figure A1:**
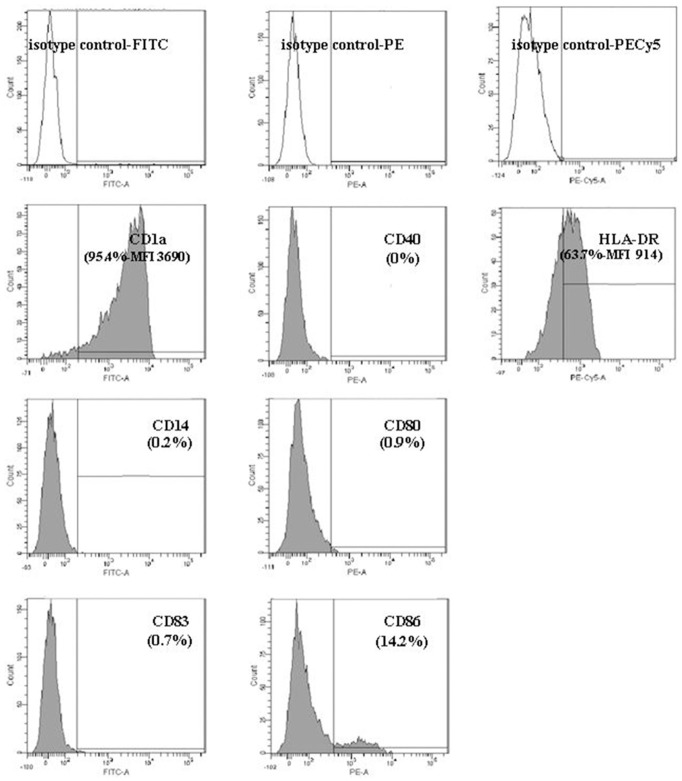
**Characterization of the immature state of dendritic cells after 7 days of differentiation with GM-CSF and IL-4 as determined by flow cytometry.** After 7 days of culture in the presence of GM-CSF and IL-4, DCs were labeled with the following antibodies: CD14-FITC, CD1a-FITC, CD40-PE, CD80-PE, CD83-FITC, CD86-PE, and HLA-DR-PECy5. They were analyzed by flow cytometry using a Canto II cytometer (Becton Dickinson, Le Pont de Claix, France). The percentages of positively labeled cells were determined after subtraction of the isotype control values (black histograms on the top of the figure) and are presented in gray histograms for each surface marker. In mature DCs, CD40, CD80, CD83, and CD86 were low in accordance with a non-activated stage. A high proportion of DCs expressed the CD1a and HLA-DR molecules. For each activation marker data [percentage of positive cells + mean fluorescence intensity (MFI)] are indicated. One representative experiment out of 13 is shown.

**Figure A2:**
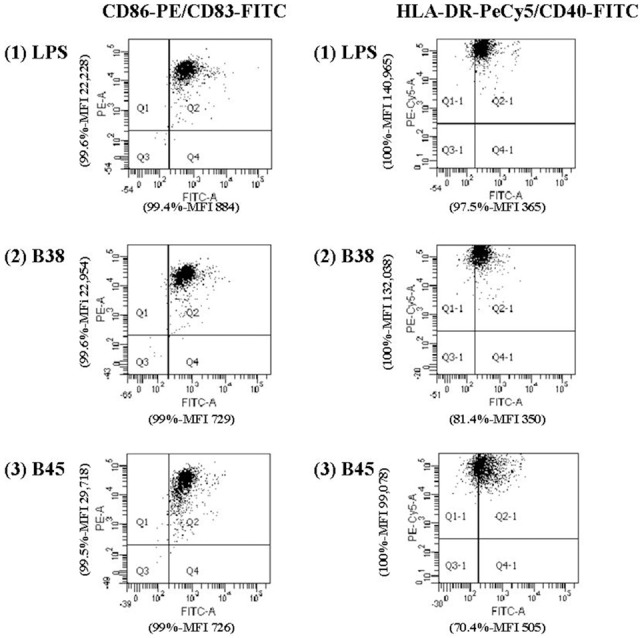
**Upregulation of HLA-DR, CD86, CD40, and CD83 expression in activated dendritic cells.** Dot plots on the left side of the figure correspond to DCs stained with C83-FITC and CD86-PE antibodies and dot plots on the right side to DCs stained with C40-FITC and HLA-DR-PECy5 antibodies. *E. coli* LPS (LPS, 100 ng/ml) was used as the positive control. For each activation marker, the percentage of positive cells and mean fluorescence intensity (MFI) are indicated on each corresponding axis and are representative of the data obtained for one experiment out of 13 performed for *H. pylori* strain B38 and strain B45. (PE: phycoerythrine, PECy5: phycoerythrine-cyanine 5, FITC: fluorescein isothiocyanate).

**Figure A3:**
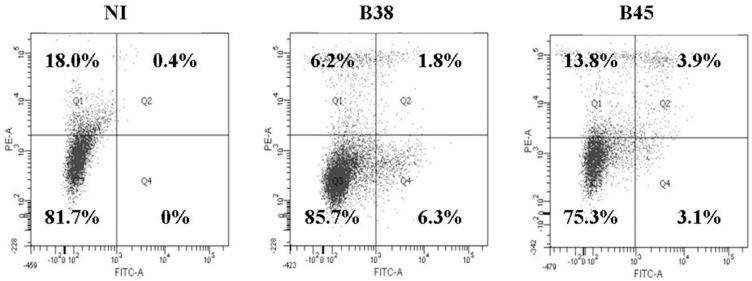
**Apoptosis rate in dendritic cell apoptosis activated by *H. pylori*.** DCs were cocultivated for 48 h with B38 or B45 *H. pylori* strain at MOI 1. The cells were labeled with anti-annexin V-FITC antibody and propidium iodure and analyzed by flow cytometry. Percentages of viable DCs (double negative cells in Q3), necrotic DCs (propidium iodure positive-FITC negative in Q1), and apoptotic DCs (FITC-positive, propidium iodure ± in Q2 and Q4) are depicted in each area. The percentages are shown for one representative experiment out of 3 independent experiments (y-axis, propidium iodure; x-axis, anti-annexin V-FITC) (NI for uninfected DCs).

The nature, the amount and the duration of the antigenic stimulation are important factors that can influence the inflammatory and molecular responses of DCs. Before infection, the immature DC phenotype (CD1a +, HLA-DR +, CD80 −, CD86 −, CD14 −, CD83−) was verified by flow cytometry (Figure A1). Our preliminary experiments showed that *H. pylori* at MOI 1 significantly activated DCs (Figure A2), with minimal apoptosis (less than 10%) (Figure A3).

### Cytokine production in *H. pylori*-activated DCs

DCs activated by either the B38 or B45 strain at MOI 1 or by *E. coli* LPS secreted significant amounts of TNFα, IL-1β, IL-6, IL-8, IL-10, IL-12, and IL-23, but not IL-2, IL-4, IL-17, TGFβ, or INFγ compared to non-activated DCs. *H. pylori*-activated DCs produced mainly TNFα (1500–2000 fold stimulated production over basal level), and in a decreasing order of stimulation, IL-6 (1000-fold), IL-10 (200–400 fold), IL-1β (150-fold), IL-23 (50–100 fold), IL-8 (10-fold) and IL-12 (5-fold) after 48 h of co-culture. The cytokine levels were low or nil in non-activated DC supernatants, except for IL-8 and IL-12 which were already secreted under basal conditions.

**Figure 1:**
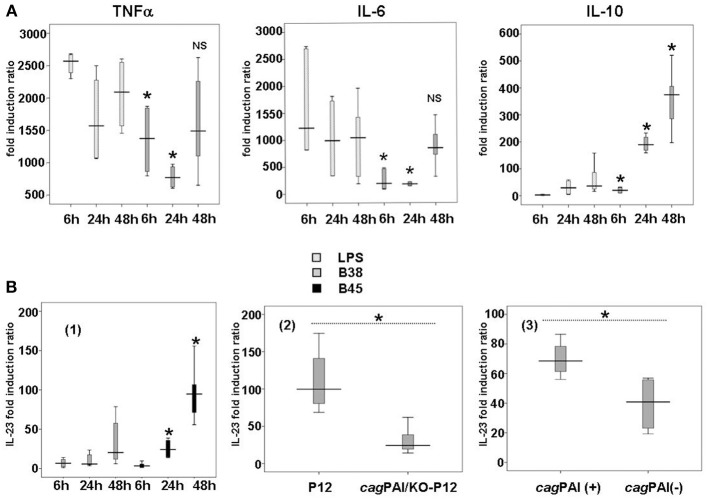
**Cytokine production in *H. pylori*-activated dendritic cells. (A)** Kinetics of TNFα, IL-6, and IL-10 secretion by *E. coli* LPS- or *H. pylori*-activated dendritic cells. DCs were either stimulated with *E. coli* LPS (100 ng/ml) or cocultivated with B38 *H. pylori* strain at MOI 1 for 6, 24, or 48 h. TNFα, IL-6, and IL-10 secretion in the culture supernantants was evaluated by flow cytometry (as described in Materials and Methods). The horizontal bar across the box indicates the median and the capped bars indicate the minimum and maximum data values of fold induction ratio vs. DCs alone obtained for 6 independent experiments corresponding to 6 different donors. These results are representative of the results obtained for *H. pylori* strain B45 for the same experiments. *for *p* < 0.05 and NS for *p* > 0.05 compared to *E. coli* LPS at the corresponding time-point. **(B)** Influence of the *H. pylori cag*PAI on the production of IL-23 by dendritic cells after *H. pylori* infection. (1) DCs were cocultured with B38 or B45 *H. pylori* strain at MOI 1 for 6, 24, or 48 h. IL-23 levels in culture supernatants were measured by ELISA. The horizontal bar across the box indicates the median and the capped bars indicate the minimum and maximum data values of fold induction ratio vs. non-activated DCs, for 6 independent experiments for each strain and for 6 different donors. *for *p* < 0.05 between the *H. pylori* strains B38 and B45 at the corresponding time-point. (2) DCs were cocultured for 48 h with *H. pylori* strain P12 and its isogenic *cag*PAI mutant (KO-P12) at MOI 1 or (3) with 12 ulcer-associated *H. pylori* strains tested individually (6 *cag*PAI positive and 6 *cag*PAI negative). IL-23 levels in culture supernatants were measured by ELISA. The horizontal bar across the box indicates the median and the capped bars indicate the minimum and maximum data values of fold induction ratio vs. non-activated DCs, for 3 independent experiments for each group of strains and for three different donors (*if *p* < 0.05).

*H. pylori*-activated DCs produced the same cytokines as LPS-activated DCs, both qualitatively and quantitatively, except for IL-10 (Figure 1). This potent anti-inflammatory molecule was found at a significantly higher level upon stimulation by both *H. pylori* B38 and B45 than by LPS. Moreover, compared to *E. coli* LPS, IL-10 production was significantly induced by *H. pylori* B38 strain as early as 6 h.

Analysis of cytokine production kinetics in *H. pylori*-activated DCs revealed the early, massive production of TNFα (Figure 1). TNFα reached its maximal level as early as 6 h in the presence of LPS or the bacteria, and remained quite stable thereafter. IL-6 was maximally stimulated by *E. coli* LPS as early as 6 h, and progressively with *H. pylori* (Figure 1).

**Figure A4:**
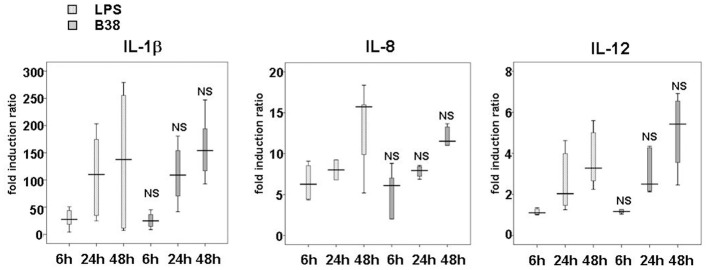
**Kinetics of IL-1β, IL-8 and IL-12 production by *E. coli* LPS- or *H. pylori*-activated dendritic cells.** DCs were either stimulated with *E. coli* LPS (100 ng/mL) or cocultivated with *H. pylori* B38 strain at MOI 1 for 6, 24, or 48 h. IL-1β, IL-8, and IL-12 secretion levels were assessed by ELISA (as described in Material and Methods). The horizontal bar across the box indicates the median and the capped bars indicate the minimum and maximum data values of fold induction ratio vs. DCs alone obtained for 6 independent experiments corresponding to 6 different donors. These results are representative of those obtained for *H. pylori* B45 strain-activated DC performed in parallel during the same experiments. NS for *p* > 0.05 compared to *E. coli* LPS-activated DC at the corresponding time-point.

There was generally no significant difference in the cytokine production between the two *H. pylori* strains, except for IL-23, a major cytokine involved in Th17 response, which was higher with B45 than with B38 (Figure 1). This difference could be attributed to the presence of the *cag*PAI in the B45 strain. To test this hypothesis, DCs were activated for 48 h by either *H. pylori* P12 strain or its isogenic *cag*PAI deletion mutant (Figure 1), and by 12 more *H. pylori* strains tested individually (6 *cag*PAI positive and 6 *cag*PAI negative) from our collection (Lehours et al., 2004a) (Figure 1). IL-23 secretion was significantly lower with the P12 mutant and the *cag*PAI negative strains than with the P12 wild type and the *cag*PAI positive strains confirming the positive influence of the *cag*PAI gene products on the secretion of this cytokine. IL-23 secretion started rather late following *H. pylori* infection, at 48 h for B38 and 24 h for B45 (Figure 1). Finally, both LPS- and *H. pylori*-mediated activation displayed similar patterns of progressive production of IL-1β, IL-8, and IL-12 over the time course (Figure A4).

### Expression of miRNA in *H. pylori*-activated DCs

MiRNAs, differentially regulated in *H. pylori*-activated DCs as compared to immature DCs, were investigated using a qPCR array of 88 miRNA involved in human immunopathology. Among the screened miRNAs, 12 showed the highest induction levels (3–21 fold) and 13 the strongest repression (3–6 fold) (Table A1). Nevertheless, taking into account only those that were expressed the most upon induction (over 1/100 of average housekeeping genes), we identified miR-187, miR-155, miR-146a, let-7e, miR-29b, miR-34, miR-214, and miR-147 as being substantially upregulated following *H. pylori* infection. The relatively abundant miR-152, miR-195, miR-16, miR-30c, miR-223, miR-126, miR-574-3p, miR-21, and miR125b were found to be the most repressed by the bacteria (Table 1).

Table 1**miRNA differentially regulated in *H. pylori* B38-activated dendritic cells**.

**Table:** 

**Up-regulated miRNA**	**Fold change upon infection**	**Relative level in infected cells**
**hsa-miR-187**	**21.0756**	**0.0515**
**hsa-miR-155**	**13.4310**	**2.5491**
hsa-miR-198	7.6079	0.0023
hsa-miR-135b	6.5319	0.0049
hsa-miR-299-3p	5.5693	0.0020
hsa-miR-451	4.8821	0.0052
**hsa-miR-146a**	**4.8149**	**21.8566**
**hsa-let-7e**	**4.7816**	**2.4284**
hsa-miR-184	4.3094	0.0016
**hsa-miR-29b**	**4.2797**	**0.1216**
**hsa-miR-34a**	**4.1339**	**0.7738**
**hsa-miR-214**	**4.1626**	**0.0110**
hsa-miR-206	3.1987	0.0046
**hsa-miR-147**	**3.0898**	**0.3276**
hsa-miR-302a	3.0262	0.0015

**Table:** 

**Downregulated miRNA**	**Fold change upon infection**	**Relative initial level**
hsa-miR-409-3p	0.1728	0.0904
**hsa-miR-152**	**0.2070**	**0.2897**
hsa-miR-150	0.2113	0.0333
**hsa-miR-195**	**0.2584**	**145.2606**
**hsa-miR-16**	**0.2675**	**202.6014**
**hsa-miR-30c**	**0.3051**	**12.6626**
**hsa-miR-223**	**0.3115**	**143.2608**
**hsa-miR-126**	**0.3248**	**1.6386**
**hsa-miR-574-3p**	**0.3270**	**1.3218**
**hsa-miR-21**	**0.3293**	**1307.4137**
hsa-miR-203	0.3362	0.0168
**hsa-mir-125b**	**0.3529**	**1.9219**

*One μg of total RNA from DCs cultured for 48 h in the absence or presence of H. pylori B38 at MOI 1 was retro-transcribed using the RT2 miRNA First Strand Kit (Qiagen, Courtaboeuf, France). The first strand reaction was mixed with RT2 SYBR Green qPCR mix and distributed onto PCR array plates containing primers for 88 miRNA involved in human immunopathology (SABioscience, Qiagen) according to manufacturer's protocol. The amplification profiles were measured on a Stratagene Mx3005P instrument (Life Technologies, Saint Aubin, France). Data were analyzed by the C_t_ method (Livak and Schmittgen, 2001), using the average C_t_ of the housekeeping RNA for normalization. Data are expressed as fold induction compared to non-activated DCs in the medium lane. For each miRNA, the level relative to the mean expression of housekeeping genes is shown in the right lane, in order to assess which miRNA may be expressed at levels high enough to be relevant (highlighted)*.

**Figure 2:**
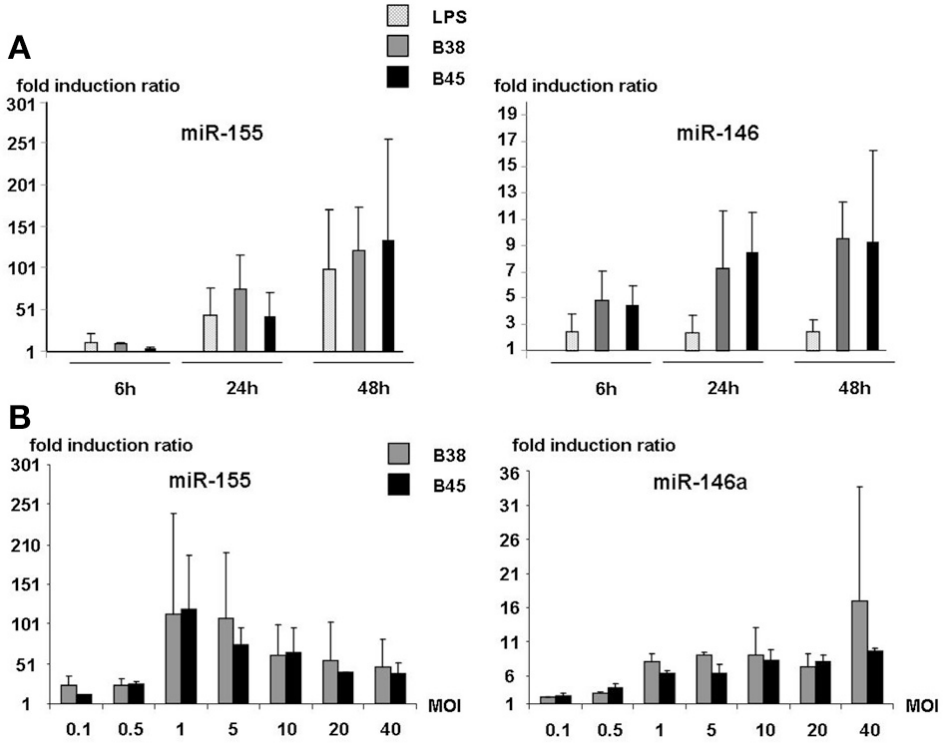
**MOI dependence and kinetic analyses of miR-146a and miR155 expression in activated dendritic cells. (A)** DCs were either stimulated with *E. coli* LPS or cocultured with *H. pylori* strains B38 or B45 at MOI 1 for 6, 24, or 48 h. **(B)** DCs were cocultured with *H. pylori* strains B38 or B45 at MOIs ranging from 0.1 to 40 for 48 h. For both experiments, miRNA levels were determined by RT-qPCR. Data is presented as the fold induction ratio (mean + standard deviation of duplicate) of activated vs. non-activated DCs and is representative of one experiment out of 3.

Kinetic analyses of miR-146 and miR-155 expression in DCs co-cultured with either the B38 or the B45 strain at MOI 1 showed that both miRNAs were upregulated as early as 6 h post-infection and increased progressively thereafter (Figure 2A). Whereas miR-146a reached 50% of its maximal stimulation as early as 6 h post-infection, miR-155 was dramatically upregulated between 6 and 24 h. Thus, at 48 h post-infection, miR-146 levels were increased by 5–10 fold over the basal level, and miR-155 by 100–200 fold. The statistical analysis of 13 independent experiments showed no significant difference between the B38 and B45 strains in their ability to upregulate the miRNAs, since miR146a expression was enhanced by 4.06 ± 0.47 and 4.8 ± 0.78 fold (*p* = 0.927), and miR-155 by 84.03 ± 15.87 and 99.17 ± 26.94 fold (*p* = 0.224), respectively. This suggests that the miRNA response of *H. pylori*-activated DC was independent of the strain's *cag*PAI status. miR-146a was upregulated more by the *H. pylori* strains than by LPS, contrary to miR-155, which reached the same intensity after LPS stimulation as after co-culture with either of the *H. pylori* strains (Figure 2A). At 48 h post-infection, miR-146a upregulation was positively correlated with a MOI up to 40, whereas miR-155 upregulation peaked at MOI 1–5 and decreased at higher ones (Figure 2B). The global results indicate that miR-146a and miR-155 upregulations take part in the DC immune response to *H. pylori*, independently of their *cag*PAI status, with miR-146a upregulation preceding the massive miR-155 biosynthesis.

**Figure 3:**
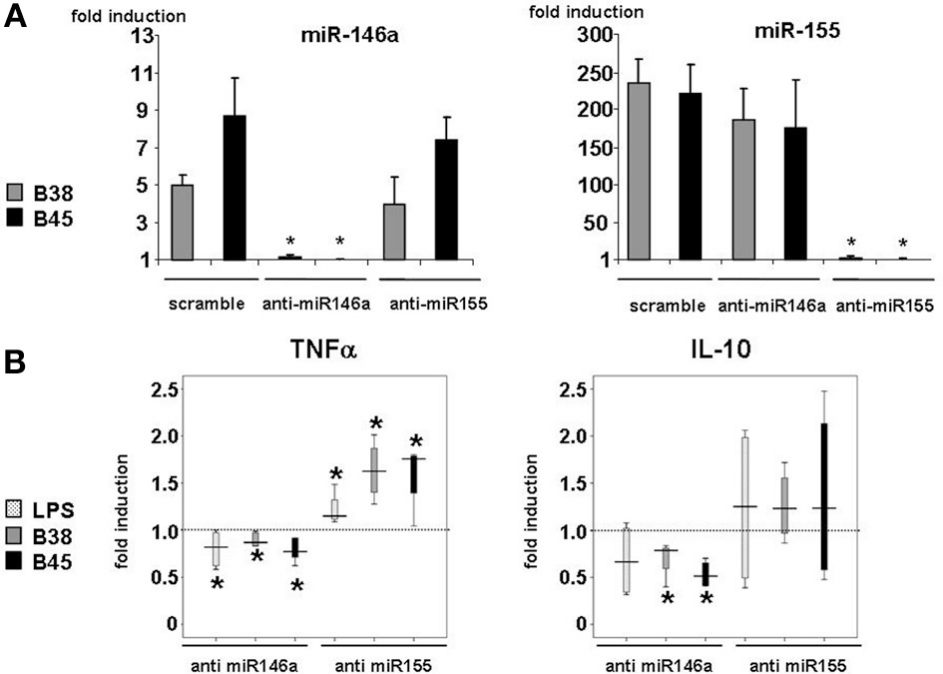
**Effect of miR-146a and miR-155 loss of function in *H. pylori*-induced miRNA levels and TNFα and IL-10 production. (A)** Changes in miR-146a and mir-155 relative levels in DCs transfected with anti-miRNA oligonucleotides: DCs were transfected with 100 nM of miRNA-antisense (anti-miR-146a or anti-miR-155) or scrambled oligonucleotides before being challenged with B38 or B45 *H. pylori* strains at MOI 1 for 48 h. RTqPCR data are presented as the fold induction ratio (mean + standard deviation of triplicates) in activated DCs and are representative of one experiment out of 4.*if *p* < 0.05 compared to miRNA levels obtained on activated DCs treated with scrambled oligonucleotides. **(B)** TNFα and IL-10 production in activated DCs transfected by either anti-miR146a or anti-miR155 oligonucleotides: DCs were transfected as in (A) and activated with *E. coli* LPS (100 ng/ml) or *H. pylori* B38 or B45 strains at MOI 1 for 48 h. TNFα and IL-10 production was evaluated by flow cytometry. The horizontal bar across the box indicates the median and the capped bars indicate the minimum and maximum data values of fold induction ratio compared to scrambled oligonucleotide-treated cells for 4 independent experiments corresponding to 4 different donors (*if *p* < 0.05).

**Figure A5:**
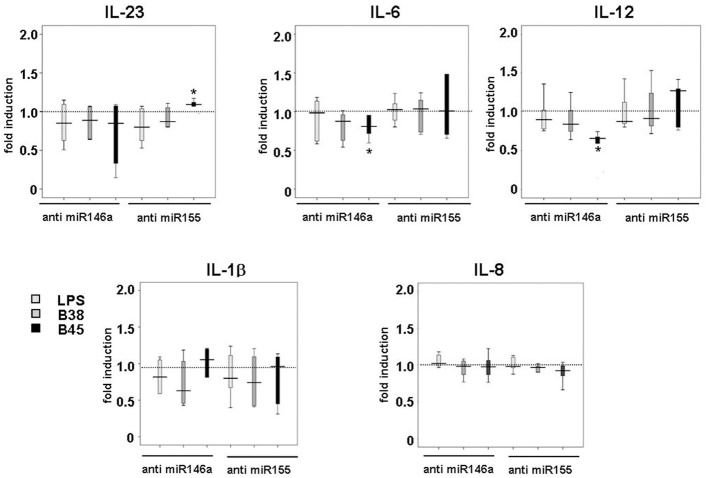
**Effect of miR-146a or miR-155 loss of function on *H. pylori*-induced IL-23, IL-6, IL-12, IL-1β and IL-8 production.** DCs were transfected with 100 nM of miRNA-antisense (anti-miR-146a or anti-miR-155) or scrambled oligonucleotides before being challenged with B38 or B45 *H. pylori* strains at MOI 1 for 48 h. IL-6 secretion was evaluated by flow cytometry, whereas IL-1β, IL-8, IL-12, and IL-23 production were assessed by ELISA. The horizontal bar across the box indicates the median and the capped bars indicate the minimum and maximum data values of fold induction ratio compared to scrambled oligonucleotide-treated cells, for 4 independent experiments corresponding to 4 different donors (*if *p* < 0.05).

In order to investigate the importance of these miRNAs in the regulation of cytokine production upon infection, their mature forms were functionally inhibited using antisense oligonucleotides (or their scrambled controls), which were introduced into immature DCs using lipofectamin prior to the LPS or *H. pylori* challenge. Both miR-146a and miR-155 knockdown had little influence on the LPS- or *H. pylori*-mediated upregulation of DC surface activation markers, suggesting that neither one was required for this process (data not shown). Anti-miR-146 antisense oligonucleotides totally prevented LPS- or *H. pylori*-triggered miR-146 upregulation (Figure 3A) and had no influence on the level of miR-155 induction, and *vice versa*, showing the efficiency of antisenses to inhibit miRNA expression, most likely by sequestering them. This also suggests that miR-146a and miR-155 expression may not be correlated in this cell system. Nevertheless, in response to *H. pylori*, two cytokines were clearly affected by this treatment (Figure 3B). First, in comparison to scrambled oligonucleotide-treated cells, TNFα production at 48 h was induced to a 50% greater extent in anti-miR-155-treated cells notably upon *H. pylori* infection, whereas it was weakly impaired (≤20%) by anti-miR-146. Second, anti-miR-146 treatment clearly impaired IL-10 production upon infection, whereas anti-miR-155 had no significant effect. miR-146a or miR-155 knockdown did not affect the cytokine response to LPS. This treatment did not alter IL-1β or IL-8 production and only weakly affected that of other cytokines after *H. pylori* challenge (Figure A5).

## Discussion

In this manuscript, we aimed at analyzing the initial steps of the immune response to *H. pylori* strains. We therefore developed an *ex vivo* co-culture cell system, in which we showed that *H. pylori* strains were able to activate primary human DCs and promote strong proinflammatory cytokine and miRNA responses. Although most of the observed effects were independent of the *H. pylori cag*PAI status, the *cag*PAI positive strain specifically upregulated IL-23, most likely to orientate DCs to promote a Th17 response.

Numerous articles have been published on the interaction between *H. pylori* and DCs, however, with a huge heterogeneity in the bacterial strains, MOI and experimental conditions (viable/sonicated/paraformaldehyde-treated bacteria) (Guiney et al., 2003; Hafsi et al., 2004; Kranzer et al., 2004, 2005; Kao et al., 2006; Mitchell et al., 2007; Rad et al., 2009; Andres et al., 2010; Kao et al., 2010; Fehlings et al., 2012). Our data were obtained using primary human DCs from several donors. All experiments were repeated with at least three different donors in order to ensure the reliability of the results. Significant productions of several cytokines (TNFα, IL-1β, IL-6, IL-8, IL-10, IL-12, and IL-23) were observed in *H. pylori*-activated DCs, TNFα and IL-6 being produced at the highest levels. *In vivo*, TNFα contributes to monocyte maturation after recruitment, whereas IL-6 supports the transition between the early stages of the infection and the sustained mononuclear influx into the infected gastric mucosa. Early production of TNFα and IL-6 upon LPS stimulation reflects the intrinsic property of *E. coli* LPS to trigger TLR4 signaling pathways, a property that is not shared by *H. pylori* LPS (Rad et al., 2009). Delayed secretion of TNFα and IL-6 by *H. pylori*-activated DCs as compared to LPS-activated ones may depend on bacterial DNA and RNA recognition by endosomal TLR9 and TLR8, respectively (Rad et al., 2009), a later process when compared to the recognition of pathogen associated-motifs by surface TLRs. At later time points (24 and 48 h), a switch in the inflammatory response occurred with an accumulation over time of IL-6, IL-10, IL-23, and IL-1β, without any significant induction of TGFβ. The late IL-10 and IL-12 production by *H. pylori*-activated DCs may coincide *in vivo* with their homing in lymph nodes (Kranzer et al., 2004). IL-10 was secreted at a higher level after DC activation by *H. pylori* than by *E. coli* LPS, in accordance with recent data, which showed that *H. pylori* interactions with both TLR2 and the lectin DC-SIGN contributed to an anti-inflammatory environment *via* the release of IL-10 (Rad et al., 2009; Fehlings et al., 2012). IL-12, which plays a role in the differentiation of naive T cells toward Th1 cells (Pellicano et al., 2007), was the least produced cytokine upon DC activation. In the present study, the high IL-10 and TNFα levels secreted by activated DCs could be responsible for the low IL-12 secretion due to their ability to inhibit its production (Langenkamp et al., 2000). In addition, IL-23 was the only cytokine dependent on the strain's *cag*PAI status. This result is still a matter of debate. Indeed several papers show that *cag*PAI could influence *in vitro* IL-23 release by DCs (Khamri et al., 2009; Tanaka et al., 2010). However, others argue that *cag*PAI does not regulate cytokine production in DCs cocultured with *H. pylori* (Kao et al., 2010; Horvath et al., 2012). Previous data from experiments on human gastric biopsies infected with *H. pylori* indicate that myeloid DCs co-localize with IL-23 and IL-17-producing, infiltrating lymphocytes (Khamri et al., 2010); one could therefore hypothesize that the *H. pylori* B45 strain may be able to induce a Th17 response, likely mediated by the *cag*PAI status. As IL-23 also plays an important role in sustaining Th17 responses in addition to acting on lymphoid cells to induce IL-17 (Shi et al., 2010; Hitzler et al., 2013), signaling pathways involved in this phenomenon deserve further investigation.

In parallel to the cytokine response profile, miR-155 and miR-146a were co-upregulated most intensely and invariably in activated DCs, independently of the *cag*PAI status of the bacterial strain. These two miRNA were previously expressed at high levels in *H. pylori*-infected gastric mucosa (Liu et al., 2010; Lario et al., 2012). Our results clearly show that the extent of their upregulation as well as their kinetics and MOI-dependence did not evolve in parallel with the time-course of *H. pylori*-activated DCs. MiR-146 upregulation takes part in the early immune program activated in DCs by *H. pylori*, as it reached 50% of its maximal 10 fold stimulation as early as 6 h post-infection, while miR-155 was induced at a striking rate 24 h post-infection, reaching 200 fold the stimulated levels at 48 h. The massive induction of miR-155 in DCs over the time-course of their activation by both LPS and *H. pylori* is consistent with previous findings (O'Connell et al., 2007; Tili et al., 2007; Martinez-Nunez et al., 2009). Indeed, miR-155 can be induced by pathogen-associated ligands via TLR-, NF-κ B-, and MyD 88-dependent pathways or by several cytokines such as IL-1β (Ceppi et al., 2009) and TNFα (Tili et al., 2007) which are both likely to sustain the high miR-155 biosynthesis induced in LPS-stimulated or *H. pylori*-infected DCs. An alternative miR-155 activation by bacterial peptidoglycan sensing via the cytoplasmic NOD2 receptor has been reported (Schulte et al., 2013), which may participate to the massive miR-155 induction. Contrary to miR-146, miR-155 was induced via a biphasic profile according to the MOI, suggesting that at a high MOI, the increasing level of some inflammatory cytokines might negatively regulate miR-155 expression. As the anti-inflammatory cytokine IL-10 was found to inhibit LPS-induced miR-155 upregulation without affecting miR-146a (McCoy et al., 2010), it is likely that *H. pylori*-stimulated IL-10 production is responsible for the decreased miR-155 upregulation at high MOIs.

The role of miR-146 and miR-155 in the innate immune responses was deduced from the identification of their respective targets. As miR-146a targets and silences TNF-receptor-associated factor and IL-1β receptor-associated kinase mRNAs, which are key adapter molecules in the TLR/ NFκ B pathway, the role of miR-146a upregulation in response to pathogens was shown to moderate TLR-triggering of the NFκ B pathway via a negative feedback loop, thus avoiding overproduction of pro-inflammatory IL-1β and TNFα cytokines (Taganov et al., 2006; Nahid et al., 2009; Lu et al., 2010). A similar immune regulatory function was assigned to miR-155 which targets several gene transcripts of the NF-κ B pathway (FADD, Ripk1, IKK, and NIK), as well as the immune cell transcription factor PU, Src homology 2 domain-containing inositol-5-phosphatase (SHIP) and CCAAT enhancer-binding protein beta (C/EBPbeta) (Rodriguez et al., 2007; Tili et al., 2007; Costinean et al., 2009; Schulte et al., 2013). In addition, miR-155 also stabilizes the TNFα mRNA (Tili et al., 2007; Semaan et al., 2011), suggesting that simultaneous anti- and pro-inflammatory effects of miR-155 most likely contribute to the fine-tuning of the TNF-mediated inflammation. We assessed the role of miR-146 and miR-155 in the DC cytokine response to *H. pylori* using specific antisenses to each miRNA, which inhibit their silencing function by competition with their binding sites on mRNA targets. Thus, the most noticeable effect of miR-155 loss of function in our cell system was an enhanced TNFα production by *H. pylori*, an effect consistent with the immunomodulatory role assigned to miR-155 upregulation in response to microbial stimuli (O'Connell et al., 2007; Rodriguez et al., 2007; Ceppi et al., 2009; Martinez-Nunez et al., 2009). Enhanced TNFα response to *H. pylori* in the absence of miR-155 may also be facilitated by the downregulation of miR-125b which we observed in our microarray (Table 1), and which directly targets TNFα (Tili et al., 2007). In our miR-146 loss of function experiment, no cytokine response was markedly altered with the exception of IL-10. The impaired production of this anti-inflammatory cytokine in response to *H. pylori* is concordant with the previously established immunomodulatory role of miR-146 upon infection, but it was not accompanied by an enhanced production of inflammatory cytokines in anti-miR-146-treated cells as expected. A possible explanation for the decreased IL-10 response to *H. pylori* in the absence of miR-146 could be that miR-146 loss of function unmasks the regulatory effects of other miRNAs: for instance, let-7e, which directly targets IL-10 (Schulte et al., 2011) was upregulated in *H. pylori*-activated DCs (Table 1) and may subsequently negatively regulate IL-10. The differences in the effects of miR-146 and miR-155 knockdown, which stress the prominent role of miR-155 in *H. pylori*-activated DCs, could be related to different functional specializations of these two seemingly co-induced miRNAs, as recently reported in LPS-stimulated murine macrophages (Schulte et al., 2013); miR-146 responded to sub-inflammatory stimuli to prevent TLR activation, and miR-155 responded to pro-inflammatory stimuli as a global limiter of the inflammatory response. Our experimental conditions established a TNFα-filled, pro-inflammatory environment in cultured DCs, and thus preferentially elicited miR-155 functions.

miR-146a and miR-155 expressions may be of special interest in *H. pylori*-mediated immune pathologies, because they are associated with NFκB activation, a pathway strongly activated during *H. pylori* infection and in *H. pylori*-driven MALT lymphoma. High expression of miR-146a was associated with chronic inflammatory diseases (Sonkoly and Pivarcsi, 2009) and miR-155 represents an important element in B cell lymphoma development (Costinean et al., 2006), indicating that alterations in the fine-tuning of innate immune responses by miRNAs may contribute to inflammatory disorders. Other miRNAs, which are differentially regulated in *H. pylori*-activated DCs, deserve further investigation; among them, miR-187, which was strongly upregulated in the *H. pylori*-activated DCs, could control IL-10-driven anti-inflammatory responses (Rossato et al., 2012).

In conclusion, the co-culture cell system presented here allowed the identification of pro- and anti-inflammatory effectors in response to *H. pylori* and revealed the influence of the *cag*PAI on the immune responses. These important findings pave the way for further investigations on the nature of *H. pylori* antigens and the subsequently activated signaling pathways involved in the inflammatory response to *H. pylori* infection.

### Conflict of interest statement

The authors declare that the research was conducted in the absence of any commercial or financial relationships that could be construed as a potential conflict of interest.

## Appendix

**Table A1:** **MiRNA microarray data in uninfected DC (control) and DCs infected for 48 h by *H. pylori* B38 strain at MOI 1**.

**Mature miRNA ID**	**Ct control DC**	**Ct B38-infected**	**Rel value in control**	**Rel value in B38-infected**	**Fold change upon infection**
hsa-let-7a	21.6	18.34	2.7368	4.3169	1.5773
hsa-let-7c	29.79	26.4	0.0094	0.0162	1.7261
hsa-let-7d	25.72	22.11	0.1574	0.3164	2.0104
hsa-let-7e	24.03	19.17	0.5079	2.4284	4.7816
hsa-let-7g	21.28	17.74	3.4165	6.5432	1.9152
hsa-miR-103	20.51	17.97	5.8260	5.5790	0.9576
hsa-miR-106a	19.89	18	8.9538	5.4642	0.6103
hsa-miR-125a-5p	19.32	17.27	13.2921	9.0631	0.6818
hsa-mir-125b	22.11	21.01	1.9219	0.6783	0.3529
hsa-miR-126	22.34	21.36	1.6386	0.5322	0.3248
hsa-miR-128	23.57	22.36	0.6986	0.2661	0.3809
hsa-miR-130a	30.73	27.14	0.0049	0.0097	1.9827
hsa-miR-132	21.35	19.8	3.2546	1.5692	0.4821
hsa-miR-135b	33.42	28.11	0.0008	0.0049	6.5319
hsa-miR-138	31.96	28.63	0.0021	0.0034	1.6558
hsa-miR-142-3p	18.17	15.83	29.4971	24.5900	0.8336
hsa-miR-142-5p	21.05	19.47	4.0069	1.9725	0.4923
hsa-miR-143	28.96	27.05	0.0167	0.0103	0.6188
hsa-miR-145	29.57	27.39	0.0109	0.0081	0.7461
hsa-miR-146a	20.87	16	4.5394	21.8566	4.8149
hsa-miR-147	26.29	22.06	0.1060	0.3276	3.0898
hsa-miR-148a	23.17	21.27	0.9218	0.5664	0.6145
hsa-miR-150	27.96	27.6	0.0333	0.0070	0.2113
hsa-miR-152	24.84	24.51	0.2897	0.0600	0.2070
hsa-miR-155	25.45	19.1	0.1898	2.5491	13.4310
hsa-miR-15a	22.35	19.39	1.6273	2.0849	1.2812
hsa-miR-15b	21.33	19.35	3.3001	2.1435	0.6495
hsa-miR-16	15.39	14.69	202.6014	54.1917	0.2675
hsa-miR-181a	25.92	22.12	0.1370	0.3143	2.2934
hsa-miR-182	29.4	27.82	0.0123	0.0060	0.4923
hsa-miR-183	32.22	29.45	0.0017	0.0020	1.1231
hsa-miR-184	34.48	29.77	0.0004	0.0016	4.3094
hsa-miR-185	23.65	21.63	0.6609	0.4414	0.6678
hsa-miR-186	24.85	21.41	0.2877	0.5141	1.7870
hsa-miR-187	31.73	24.73	0.0024	0.0515	21.0756
hsa-miR-18a	21.9	20.46	2.2230	0.9931	0.4467
hsa-miR-18b	28.4	25.7	0.0246	0.0263	1.0699
hsa-miR-191	18.39	17.18	25.3252	9.6465	0.3809
hsa-miR-194	25.03	23.52	0.2539	0.1191	0.4689
hsa-miR-195	15.87	15.22	145.2606	37.5307	0.2584
hsa-miR-196a	32.29	29.02	0.0017	0.0026	1.5883
hsa-miR-198	34.73	29.2	0.0003	0.0023	7.6079
hsa-miR-19a	18.9	16.6	17.7839	14.4200	0.8108
hsa-miR-19b	19.13	16.88	15.1632	11.8762	0.7832
hsa-miR-200a	31.24	29.64	0.0034	0.0017	0.4991
hsa-miR-203	28.95	27.92	0.0168	0.0056	0.3362
hsa-miR-205	31.25	30.96	0.0034	0.0007	0.2013
hsa-miR-206	32.49	28.21	0.0014	0.0046	3.1987
hsa-miR-20a	19.34	17.42	13.1091	8.1681	0.6231
hsa-miR-20b	20.8	19.06	4.7651	2.6208	0.5500
hsa-miR-21	12.7	11.7	1307.4137	430.5390	0.3293
hsa-miR-210	25.13	21.08	0.2369	0.6462	2.7274
hsa-miR-214	31.61	26.95	0.0027	0.0110	4.1626
hsa-miR-223	15.89	14.97	143.2608	44.6318	0.3115
hsa-miR-23b	22.38	20.89	1.5938	0.7371	0.4625
hsa-miR-26a	18.23	16.05	28.2955	21.1121	0.7461
hsa-miR-26b	18.8	16.95	19.0603	11.3137	0.5936
hsa-miR-27a	17.31	15.27	53.5383	36.2523	0.6771
hsa-miR-27b	22.41	20.95	1.5610	0.7071	0.4530
hsa-miR-299-3p	34.48	29.4	0.0004	0.0020	5.5693
hsa-miR-29b	28.19	23.49	0.0284	0.1216	4.2797
hsa-miR-29c	23.17	19.64	0.9218	1.7532	1.9020
hsa-miR-302a	34.03	29.83	0.0005	0.0015	3.0262
hsa-miR-30b	19.66	18.3	10.5013	4.4383	0.4226
hsa-miR-30c	19.39	18.5	12.6626	3.8637	0.3051
hsa-miR-30e	22.43	19.82	1.5395	1.5476	1.0052
hsa-miR-335	28.9	27.56	0.0174	0.0072	0.4168
hsa-miR-34a	25.47	20.82	0.1872	0.7738	4.1339
hsa-miR-363	25.96	24.12	0.1333	0.0786	0.5895
hsa-miR-370	26.25	24.36	0.1090	0.0665	0.6103
hsa-miR-409-3p	26.52	26.45	0.0904	0.0156	0.1728
hsa-miR-451	32.92	28.03	0.0011	0.0052	4.8821
hsa-miR-493	31.14	29.19	0.0037	0.0023	0.6362
hsa-miR-574-3p	22.65	21.66	1.3218	0.4323	0.3270
hsa-miR-9	25.47	22.79	0.1872	0.1975	1.0552
hsa-miR-98	24.74	22.01	0.3105	0.3392	1.0924
hsa-miR-99b	21.24	19.79	3.5125	1.5801	0.4498
SNORD48	26.92	23.15			
SNORD47	22.99	20.79			
SNORD44	19.91	18.54			
RNU6-2	22.39	19.32			
HKG mean Ct	23.0525	20.45			

*One μg of total RNA from DCs cultured for 48 h in the absence or presence of H. pylori B38 at MOI 1 was retro-transcribed using the RT2 miRNA First Strand Kit (Qiagen, Courtaboeuf, France). The first strand reaction was mixed with RT2 SYBR Green qPCR mix and distributed onto PCR array plates containing primers for 88 miRNA involved in human immunopathology (SABioscience, Qiagen) according to the manufacturer’s protocol. The amplification profiles were measured on a Stratagene Mx3005P instrument (Life Technologies, Saint Aubin, France). The relative value for each miRNA was calculated by the Δ Ct method taking account the mean Ct for housekeeping genes (HKG). The fold induction of each miRNA corresponds to the ratio of its relative value in B38-infected DCs versus its relative value in control DCs*.
